# A simplified online adaptive workflow for long-course magnetic resonance-guided radiotherapy in esophageal cancer^☆^

**DOI:** 10.1016/j.phro.2025.100717

**Published:** 2025-01-28

**Authors:** Koen M. Kuijer, Roel Bouwmans, Lando S. Bosma, Stella Mook, Gert J. Meijer

**Affiliations:** Department of Radiotherapy, University Medical Centre Utrecht, Utrecht, the Netherlands

**Keywords:** Esophageal cancer, MR-guided radiotherapy, MR-linac, Online adaptive radiotherapy

## Abstract

**Background and Purpose:**

Online adaptive magnetic resonance-guided radiotherapy (MRgRT) enables high-precision radiotherapy for esophageal cancer patients but is less feasible due to long on-table times in combination with long-course treatment. In this study, we conducted an in-silico assessment of a simplified online adaptive workflow, Adapt-To-Shape-lite (ATS-lite), in which deformable propagated contours are not modified, and assessed its feasibility.

**Materials and Methods:**

The ATS-lite workflow was simulated for all fractions of nine esophageal cancer patients who had previously received full online adaptive MRgRT with manual contour corrections if needed. The deformable propagated contours were not adjusted. A dose of 41.4 Gy in 23 fractions was prescribed. Intra- and interfraction dose accumulation were performed to evaluate target coverage per fraction and across the entire treatment. For individual fractions, coverage of the manually corrected clinical target volume (CTV) was considered adequate if V95% > 98 % and V90% > 99.5 %. Feasibility was assessed by recording treatment times in the first patients treated with ATS-lite.

**Results:**

The ATS-lite workflow provided adequate target coverage over the entire treatment for all patients, with sufficient coverage in 90% of the 177 fractions analyzed. Closer inspection revealed that inadequate target coverage in individual fractions was primarily attributed to enlargement of the manually corrected CTV, rather than poor contour propagation in the ATS-lite workflow. In seven patients, the ATS-lite workflow achieved a median time per fraction of 23 min.

**Conclusions:**

The ATS-lite workflow provides adequate target coverage and is feasible for online adaptive MRgRT in long-course esophageal cancer treatments.

## Highlights

• In the proposed simplified adaptive workflow, propagated contours are not adjusted.
• The simplified workflow provides adequate target coverage.
• The simplified workflow is feasible within 30-minute time slots.
• The simplified workflow is well-tolerated by esophageal cancer patients.
• The simplified workflow enables online adaptive treatments for esophageal cancer.

## 1 Introduction

Since the publication of the landmark CROSS trial in 2012 [1], neoadjuvant chemoradiotherapy has become a cornerstone in the curative treatment of esophageal cancer in much of the western world. The radiotherapy treatment of 23 fractions is typically delivered under cone beam CT (CBCT) guidance. Substantial interfraction variation and limited CBCT visualization of the target volume necessitates a large expansion of the clinical target volume (CTV), typically around 10 mm [2], [3], to create the planning target volume (PTV). This results in large PTVs encompassing significant amounts of healthy tissue.

Magnetic resonance-guided radiotherapy (MRgRT) using a Magnetic Resonance Linear Accelerator (MR-linac) offers potential advantages over conventional CBCT-guided radiotherapy for esophageal cancer patients. Firstly, magnetic resonance imaging (MRI) provides excellent visualization of the target volume due to its superior soft tissue contrast, enabling online adaptive protocols to resolve the interfraction variation. A significant decrease in dose to organs at risk (OARs) can be achieved by reducing the PTV margin using an online planning procedure [4].

Secondly, MR-linacs have the potential to further increase the treatment accuracy by capturing intrafraction motion. Cine-MR can track the respiratory motion and drift during treatment delivery, allowing for motion management strategies such as gating and tracking [5]. These techniques will improve treatment accuracy, thereby enabling further reduction of the CTV-to-PTV margins and sparing of the OARs.

Thirdly, MRgRT enables strategies that might increase treatment efficacy. Reduced PTV margins allow for dose escalation regimes without increasing OARs dose levels relative to CBCT-guided treatments [4]. In addition, online diffusion-weighted imaging (DWI) could potentially be used for smart dose painting, as changes in DWI signal have been shown to be a biomarker for treatment response [6], [7].

However, MRgRT also exhibits some disadvantages, including longer session times due to online imaging and replanning. Additionally, the need for onsite recontouring by a radiation oncologist adds logistical complexities. These disadvantages are even more challenging within esophageal cancer treatment. The esophageal target volume is large, and its definition requires recontouring of the gross tumor volume (GTV) as well as regeneration and adaptation of the CTV. As a result, recontouring is more labor- and time-intensive compared to regular online adaptive MRgRT treatments with smaller targets and GTV-to-PTV concepts. Moreover, the prolonged fraction durations are more challenging for long-course regimens compared to commonly used hypofractionated treatments.

A previous feasibility study demonstrated that online adaptive MRgRT for esophageal cancer reduces doses to the OARs compared to conventional CBCT-guided radiotherapy [8]. However, the treatment was only moderately feasible due to long session times, with a median of 53 min. Some patients required treatment fractions on a conventional CBCT-linac due to discomfort, and the workflow was demanding for hospital logistics and staff. Contour adaptation, with a median time of 19 min, was the most time-consuming part of the workflow.

To shorten the patient on-table time, we propose a simplified online adaptive workflow, termed Adapt-To-Shape-lite (ATS-lite), in which the deformable propagated contours are not adjusted. As a result, session times can be significantly reduced and the presence of a radiation oncologist onsite is no longer required. In this study, we performed an in-silico assessment to evaluate the dosimetric impact of the ATS-lite workflow compared to a fully adaptive workflow with manual contour adjustments. Additionally, we assessed the feasibility of the ATS-lite workflow for long-course MRgRT in esophageal cancer.

## 2 Materials and Methods

### 2.1 Patients

This study included nine esophageal cancer patients treated between July 2019 and January 2021 in a feasibility study [8]. All patients underwent chemoradiation on a 1.5 T MR-linac (Unity, Elekta AB, Stockholm, Sweden) and consented to the MOMENTUM study [9] (NCT04075305), approved by the Medical Research Ethics Committee of the University Medical Centre Utrecht in the Netherlands. Of these patients, eight received 23 fractions, and one received 28 fractions. Tumor characteristics can be found in Supplementary Materials A.

### 2.2 Pre-treatment

#### 2.2.1 Imaging

Pre-treatment MR-scans were performed on a 1.5 T Philips Ingenia MRI scanner (Philips Medical Systems, Best, Netherlands). Patients were scanned in a head-first, supine treatment position with arms down, on a flat table top under free breathing conditions. A 3D T2-weighted MRI (0.59 × 0.59 × 2 mm^3^, TE = 87.5, TR = 1300 ms) was acquired. Additionally, a planning (^18^F-FDG PET)-4DCT was performed.

#### 2.2.2 Delineations

Structures were previously delineated as part of the full adaptive ATS workflow of each patient [8]. In short, the GTV was delineated on the 3D T2-weighted MRI, with the co-registered PET and CT images providing additional guidance. The CTV was determined by extending the GTV of the primary tumor cranio-caudally with 3 cm along the gastroesophageal tract (2 cm caudal extension in cases where the CTV extended into the stomach) and radially with 5 mm margin, while respecting anatomical boundaries. Pathological lymph nodes were included in the CTV with a 5 mm isotropic margin.

#### 2.2.3 PTV margins

A previous study demonstrated that a CTV-to-PTV margin of 2 mm in axial and 5 mm in cranial-caudal direction was adequate to absorb the intrafraction motion [4]. To account for uncertainties in propagated target structures, we used a slightly more conservative PTV margin recipe. The CTV was segmented into cranial and caudal segments based on the esophageal hiatus. The cranial CTV was expanded with 3 mm in axial and 6 mm in cranial-caudal direction, while a 6 mm isotropic margin was applied to the caudal CTV. A larger PTV margin for the CTV below the diaphragm was chosen because tumors located below the diaphragm exhibit larger breathing motion and positional variability than intrathoracic tumors [3], [10], [11]. Additionally, boundaries in the abdominal region are less distinct compared to the thoracic region, potentially increasing uncertainty in target propagation.

#### 2.2.4 Planning

A pre-treatment step-and-shoot intensity-modulated radiotherapy plan was generated using the Monaco treatment planning system version 6.2 (Elekta AB, Stockholm, Sweden), which incorporates the 1.5 T magnetic field. Plan parameters included 9 beam angles, up to 100 segments, a 4 mm grid size, and 1.0 % statistical uncertainty. The relative electron densities for lungs, trachea, main bronchi, and bony tissue were adapted from the planning CT, while the remaining body tissue density was set to 1.01 g/cm^3^. A dose of 41.4 Gy in 23 fractions was prescribed to the PTV while minimizing OARs doses (Table 1).

**Table 1:** Dosimetric parameters and objectives for the pre-treatment step-and-shoot intensity-modulated plans with a dose of 41.4 Gy in 23 fractions. The dose to the healthy structures was minimized until a PTV V95% coverage between 98.0 % and 98.5 % was achieved.

**Structure**	**Dosimetric parameter**	**Objective (target)**
PTV	V107% (44.30 Gy)	< 2 cm^3^
	V95% (39.33 Gy)	> 98 %
	V90% (37.26 Gy)	> 99 %
CTV	V95% (39.33 Gy)	100 %
Lungs	V20Gy	< 30 %
	V5Gy	< 75 %
	Mean dose	< 16 Gy
Heart	V40Gy	< 30 %
	Mean dose	< 26 Gy
Kidneys	V20Gy	< 33 %
Spleen	Mean dose	< 20 Gy

Abbreviations; PTV: planning target volume; CTV: clinical target volume. V107%, V95%, V90%: the volume which receives at least 107 %, 95 % and 90 % of the prescribed dose, respectively; V40Gy, V20Gy, V5Gy: volume which receives 40, 20 and 5 Gy, respectively.

### 2.3 Workflow simulation

The ATS-lite workflow was simulated in Monaco for each treatment fraction. First, the pre-treatment MRI was rigidly registered to the online 3D T2-weighted MRI, acquired using the same parameters as the pre-treatment scan. Subsequently, all contours were propagated onto the online MRI using deformable registration. Unlike a typical ATS workflow, no contours were adjusted. Instead, a treatment plan was directly optimized based on the unadjusted contours.

To simulate the standard full-ATS workflow, the structures previously delineated during the clinical ATS procedure were used. In this procedure, structures of the reference plan were deformably propagated onto the online MRI and manually corrected by a radiation oncologist if needed [8].

For both workflow simulations, the PTV margins described in Section 2.2.3 were applied and treatment plans were optimized using the pre-treatment planning objectives and constraints without any modification.

### 2.4 Intrafraction dose accumulation

**Fig. 1:**
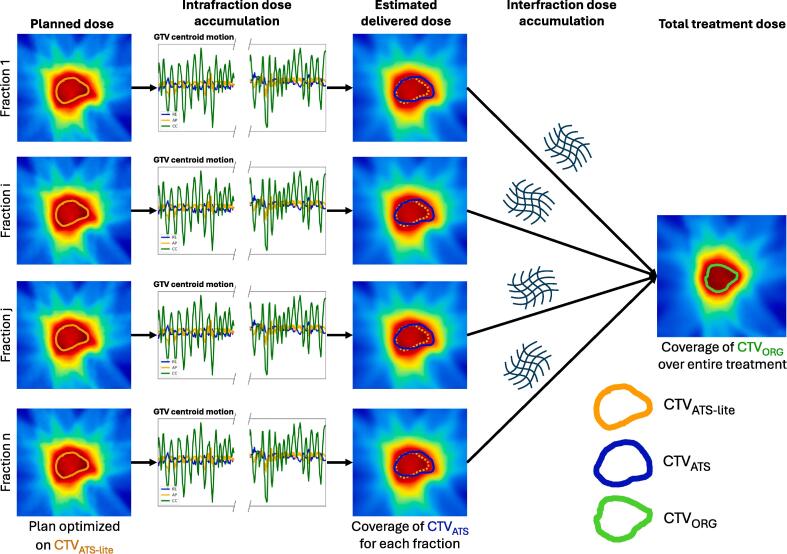
Schematic overview of the in-silico assessment for the ATS-lite workflow. For each fraction, the clinical target volume of the ATS-lite workflow simulation (CTV_ATS-lite_), expanded with the planning target volume margin, was used for treatment plan optimization. The planned dose distribution was convolved with the 3D tumor motion trajectory to derive the estimated delivered dose distribution. Individual fraction coverage by the estimated delivered dose distribution was evaluated for the manually corrected CTV during the clinical ATS procedure of that fraction (CTV_ATS_). Interfraction dose warping to the pre-treatment reference scan was performed to estimate the total treatment dose. Target coverage across the entire treatment was evaluated based on the pre-treatment CTV (CTV_ORG_). The full-ATS workflow simulation followed the same methodology, except for the evaluation of CTV_ATS_ coverage in individual fraction.

The planned dose distributions were convolved with 3D GTV motion trajectories derived from interleaved coronal and sagittal T2-weighted cine MRIs to estimate the delivered dose distributions by incorporating the intrafraction motion (Fig. 1). The cine MRIs were acquired during treatment delivery with a frequency of 3 Hz and an in-plane resolution of 1.2 mm x 1.2 mm. Each cine image was deformably registered to the reference cine frame using EVolution [12]. The resulting motion fields were applied to the GTV mask to calculate the tumor motion trajectory during beam-on time. The GTV centroid displacement in cranio-caudal and left–right directions was determined from analysis of the coronal plane, while the anterior-posterior motion was obtained from the sagittal slices. The average tumor displacement in the first 50 cine images was subtracted from all displacements to obtain motion relative to the average starting position. For each cine, the planned dose was rigidly shifted in the opposite direction of the GTV centroid displacement. All shifted dose distributions were averaged to calculate the estimated delivered dose distribution.

### 2.5 Interfraction dose accumulation

The estimated delivered dose distribution of each fraction was non-rigidly warped to the pre-treatment MRI (Fig. 1). This process involved a rigid registration followed by non-rigid registration using EVolution [12] to align the voxels from the online MRI to the pre-treatment scan. The in-house developed EVolution registration algorithm employs a multi-modal data-fidelity term that aligns image intensity gradients and a smoothness regularization that ensures that nearby voxels transform in a similar fashion. We use a regularization parameter of α = 0.3. The algorithm uses a coarse-to-fine scheme, starting iterations on the 16-fold downsampled images and upsampling with factors of two. The rigid transformation matrix and non-rigid deformation vector field were applied to the fraction estimated delivered dose distribution. Finally, for both the ATS-lite and full-ATS workflows, all warped dose distributions were summed and normalized by the total number of fractions to obtain the total treatment dose for each patient. Details regarding quality assurance (QA) on the registration results are provided in Supplementary Materials B.

### 2.6 Evaluation

For each patient, we assessed the coverage of the pre-treatment CTV (CTV_ORG_) by the total treatment dose for both the ATS-lite and the full-ATS workflows (Fig. 1). Additionally, for each individual fraction, we assessed the coverage of the manually corrected CTV from the clinical ATS workflow (CTV_ATS_) by the ATS-lite estimated delivered dose (Fig. 1). Target coverage of individual fractions was considered adequate if both CTV_ATS_ V95% > 98 % and CTV_ATS_ V90% > 99.5 %. Finally, heart and lungs doses from the full-ATS and ATS-lite estimated delivered dose distributions were compared for each fraction.

Of the 183 completed MR-linac fractions, 177 were successfully analyzed. Five fractions were excluded due to cine MR field-of-view misplacement or excessive out-of-plane motion, which precluded determination of tumor motion. Additionally, one fraction was excluded due to a Monaco error that necessitated patient relocation.

### 2.7 First clinical experience with the ATS-lite workflow

Workflow feasibility was evaluated by including the first patients treated with the ATS-lite workflow from October 2023 to September 2024. Esophageal cancer patients undergoing long-course chemoradiation were eligible for ATS-lite if the CTV length was within 18 cm and they met MRI safety criteria. All patients consented to the MOMENTUM study [9].

The pre-treatment phase followed the methodology outlined in Section 2.2. The ATS-lite workflow was conducted on a 1.5 T MR-Linac (Unity, Elekta AB, Sweden). After deformable contour propagation onto the online 3D T2-weighted MRI, radiation therapists (RTTs) were instructed to only correct gross evident errors when the target volume was outside the PTV. Thanks to the faster workflow, treatment delivery started without position verification. Following each fraction, an offline QA was performed to assess the propagated contours and drift, using a post-MRI acquired simultaneously with irradiation.

RTTs recorded the total on-table time and duration of all workflow steps. Median durations were calculated across all fractions. Patient tolerability was assessed by the percentage of treatment fractions delivered using the ATS-lite workflow. Interfraction dose accumulation was conducted to assess target coverage as detailed in Section 2.5.

## 3 Results

The in-silico assessment demonstrated that the ATS-lite workflow provided adequate target coverage for all patients throughout the entire treatment (Table 2). Furthermore, the ATS-lite workflow achieved comparable coverage to a full-ATS workflow, with similar heart and lung doses in both workflows (Supplementary Materials C).

**Table 2:** Target coverage achieved by the ATS-lite workflow in the in-silico assessment. The table presents the coverage of the pre-treatment CTV (CTV_ORG_) by the total treatment dose for both the ATS and the ATS-lite workflows. Additionally, it includes the number of fractions meeting the criteria for adequate coverage of the manually corrected CTV (CTV_ATS_), defined as achieving both V95% > 98 % and V90% > 99.5 %, as well as the median, 25th (p25), and 75th (p75) percentile of the dose-volume histogram metrics.

	**Total treatment**		**Individual fractions**					
	**CTV_ORG_ V95% (%)**			**CTV_ATS_ V95%**		**CTV_ATS_ V90%**		
**Patient**	**ATS**	**ATS-lite**	**Total fractions**	**Fractions > 98 %**	**Median (%) (p25-p75)**	**Fractions > 99.5 %**	**Median (%) (p25-p75)**	**Adequate coverage**
1	100	100	23	23 (100 %)	99.9 (99.8–100)	23 (100 %)	100 (100–100)	23 (100 %)
2	99.9	100	23	22 (96 %)	99.6 (99.2–99.9)	22 (96 %)	99.9 (99.8–100)	22 (96 %)
3	100	100	23	11 (48 %)	97.7 (96.4–99.5)	11 (48 %)	99.5 (99.1–100)	10 (43 %)
4	100	100	20	20 (100 %)	100 (99.9–100)	20 (100 %)	100 (100–100)	20 (100 %)
5	99.9	100	16	16 (100 %)	100 (99.9–100)	16 (100 %)	100 (100–100)	16 (100 %)
6	100	100	10	10 (100 %)	99.9 (99.9–100)	9 (90 %)	100 (100–100)	9 (90 %)
7	100	100	18	18 (100 %)	100 (99.9–100)	18 (100 %)	100 (100–100)	18 (100 %)
8	99.9	100	24	23 (96 %)	100 (99.9–100)	22 (92 %)	100 (100–100)	22 (92 %)
9	99.1	99.9	20	20 (100 %)	100 (100–100)	20 (100 %)	100 (100–100)	20 (100 %)
Total	−	−	177	163 (92 %)	99.9 (99.7–100)	162 (92 %)	100 (100–100)	160 (90 %)

**Fig. 2:**
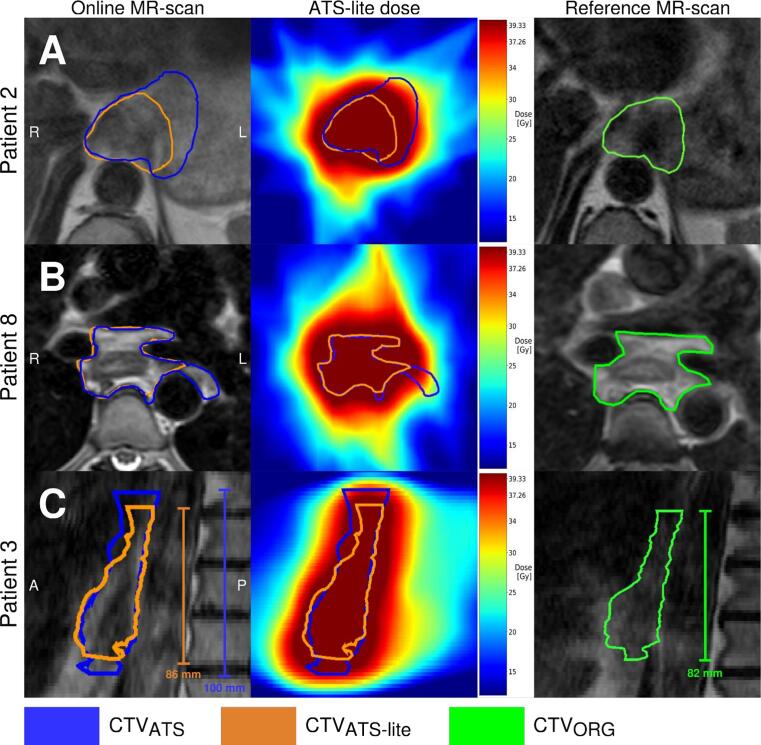
Examples of fractions with inadequate coverage of the clinical target volume (CTV) defined in the clinical ATS procedure (CTV_ATS_) for patients 2 (top), 8 (middle), and 3 (bottom). For each fraction, the unadjusted propagated CTV on which the treatment plan was optimized (CTV_ATS-lite_, orange) and the CTV_ATS_ (blue) are depicted on the online MRI (left) and the estimated delivered dose distribution (middle). The reference MRI (right) shows the reference CTV used for deformable contour propagation (CTV_ORG_, green). Notable differences between the CTV_ORG_ and the CTV_ATS_ can be observed for all three fractions.

For individual fractions, the ATS-lite workflow achieved adequate coverage of the CTV_ATS_ in 90 % of the fractions (Table 2). Closer inspection revealed that underdosage was mainly due to substantial enlargement of the CTV_ATS_ contours relative to the CTV_ORG_ on the reference MRI (Fig. 2 and Supplementary Materials D). Particularly in patient 3, target coverage was inadequate in 12 of 23 fractions, with a median CTV_ATS_ V95% of 97.7 % (range: 90.5 % − 100 %). In half of this patient’s fractions, the longitudinal CTV_ATS_ length was increased by at least 6 mm relative to the CTV_ORG_ (Supplementary Materials D), causing frequent underdosing of the CTV_ATS_ in the most cranial and caudal slices (Fig. 2C).

**Fig. 3:**
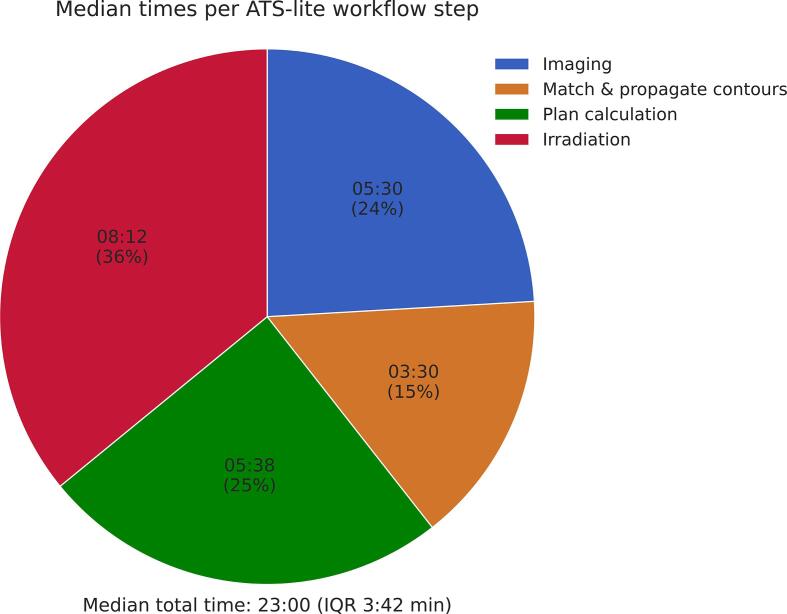
Median duration of each step in the ATS-lite workflow for online adaptive MR-guided radiotherapy for esophageal cancer patients (n = 132 fractions). IQR: interquartile range.

Seven esophageal cancer patients completed chemoradiation with the ATS-lite workflow on an MR-linac until September 2024. Of these patients, four received 23 fractions and three received 28 fractions. Tumor characteristics can be found in Supplementary Materials A. All 176 fractions were completed successfully, with no interventions necessary based on offline QA. Treatment times were recorded in 132 of the 176 fractions (Fig. 3). The median total time per fraction was 23:00 min (interquartile range (IQR) 3:42 min), with a median time of 03:30 min (IQR 01:06 min) between MRI matching and start of plan calculation. Complete target coverage was achieved for all patients.

## 4 Discussion

In this study, we demonstrated that a simplified online adaptive workflow, without manual contour adjustments, provides adequate target coverage for esophageal cancer treatment. The ATS-lite workflow was feasible, with a median total treatment time of 23 min, and well tolerated. These results indicate that this workflow can be safely implemented to facilitate online adaptive MRgRT for long-course chemoradiation in esophageal cancer.

Although 10 % of the fractions in the in-silico assessment achieved inadequate coverage of the CTV_ATS_, no underdosage of the CTV_ORG_ occurred in the total treatment dose. Closer inspection revealed that insufficient target coverage in individual fractions was primarily due to substantial enlargement of the CTV_ATS_ by the radiation oncologist during the clinical ATS workflow. This enlargement was relative to the CTV_ORG_ on the reference MRI used for contour propagation (Fig. 2 and Supplementary Materials D). This may result from a tendency to enlarge the CTV in case of doubt, especially in an online setting with time constraints. This uncertainty holds particularly for the cranial and caudal extents of the CTV, as the cranio-caudal boundary of the GTV is difficult to define without the aid of additional information such as PET-CT and endoscopy [13].

The majority of underdosed fractions occurred in patient 3, likely due to large variation in CTV_ATS_ length and substantial intrafraction motion. Radiation oncologists increased the CTV length by at least 6 mm in half of the fractions, with a maximum increase of 18 mm (Supplementary Materials D), which seems clinically unrealistic. Patient 3 also exhibited large intrafraction motion, with peak-to-peak breathing motion up to 18.3 mm and drift motion up to 10 mm in cranio-caudal direction, as reported in a previous study [14]. Nevertheless, the total treatment dose of the ATS-lite workflow achieved 100% coverage of the CTV_ORG_.

The median on-table time of patients treated with the ATS-lite workflow was 23 min. This represents a significant reduction compared to the 49.5 min reported for the standard ATS workflow [8]. The largest time savings were achieved between the end of the first MRI and the start of plan calculation, which was shortened by 18 min. Additionally, we implemented a faster MRI (3:05 min) and acquired the post-MRI during irradiation. These improvements make the ATS-lite workflow feasible within 30-minutes time slots, supporting its use for MRgRT in long-course esophageal cancer treatments.

This study demonstrated that the ATS-lite workflow achieved complete target coverage with a 6 mm isotropic CTV-to-PTV margin, except for a 3 mm axial margin above the esophageal hiatus. This margin reduction is expected to further reduce OAR doses, especially to the heart and lungs, compared to the 6 mm isotropic PTV margin applied by Boekhoff et al. [8]. Increased heart and lung doses correlate with cardiac and pulmonary complications and mortality [15], [16], [17], [18], [19], driving research into proton therapy for esophageal cancer to reduce exposure to these critical organs [20], [21], [22]. The MR-guided ATS-lite workflow provides a feasible alternative to proton therapy for sparing the heart and lungs, with potential for further margin reduction given the high target coverage and inclusion of intrafraction motion in the in-silico assessment.

Although complete target coverage was achieved for all patients, integrating offline QA to monitor intrafraction motion and contour quality is recommended. Intrafraction tumor motion can vary substantially between patients [10], [23]. Furthermore, cases of inadequate contour propagation could have been missed in this study due to the small patient cohort. The necessity for offline QA can be reassessed once online motion management strategies are in place and contour quality has been evaluated in a larger population.

The ATS-lite workflow holds promise for broader applications, particularly in long-course treatments where small propagation errors have minimal dosimetric impact and reducing on-table time is essential. For example, a simplified workflow involving rigid propagation of contours has previously been described for head and neck cancer [24]. Another potential application is in long-course rectal cancer treatment, though greater interfraction variability may pose a challenge for contour propagation.

A few limitations should be acknowledged. First, dose distributions based on the deformable propagated contours in Monaco were evaluated using interfraction dose accumulation based on deformable image registration. This could have introduced a bias in favor of the ATS-lite workflow. To minimize this, we used an independent deformable image registration algorithm for dose accumulation. Additionally, the registration quality was thoroughly verified through quantitative metrics and visual inspection, particularly for fractions with insufficient CTV_ATS_ coverage. Furthermore, as demonstrated in Supplementary Materials B, this evaluation is robust to small potential errors during interfraction dose accumulation.

Secondly, the criteria for adequate target coverage in individual fractions are somewhat arbitrary and may be considered relatively low. However, the complete target coverages across the entire treatment demonstrate that slight underdosages in single fractions are likely compensated in subsequent fractions, especially given the low doses in a long-course regimen. Additionally, intrafraction motion is incorporated in the dosimetric parameters in this study, unlike standard plan assessments. Therefore, we find these criteria acceptable for assessing target coverage in individual fractions.

Lastly, the ATS-lite workflow was simulated in an in-silico environment. Residual errors that occur during treatment delivery were not taken into account. However, these errors are very small for the ring-based 1.5 T MR-linac systems [25], [26].

In conclusion, this study demonstrated that the ATS-lite workflow provides adequate target coverage and comparable doses to the heart and lungs compared to a full online adaptive workflow. This RTT-led workflow can be safely implemented within 30-minute time slots, improving the feasibility of long-course online adaptive MR-guided radiotherapy for esophageal cancer by reducing fraction times and eliminating the need for the onsite presence of a radiation oncologist.

## CRediT authorship contribution statement

**Koen M. Kuijer:** Conceptualization, Methodology, Software, Validation, Formal analysis, Investigation, Data curation, Writing – original draft, Visualization, Project administration. **Roel Bouwmans:** Investigation, Resources, Writing – review & editing. **Lando S. Bosma:** Software, Investigation, Resources, Writing – review & editing. **Stella Mook:** Conceptualization, Supervision, Writing – review & editing, Project administration, Funding acquisition. **Gert J. Meijer:** Conceptualization, Supervision, Writing – review & editing, Project administration, Funding acquisition.

## Funding

This research was funded by the Dutch Cancer Society (KWF), project number 14388. The funding source had no role in the study design or publication of this research.

## Declaration of competing interest

The authors declare that they have no known competing financial interests or personal relationships that could have appeared to influence the work reported in this paper.
