# Supplementary material for: A simplified online adaptive workflow for long-course magnetic resonance-guided radiotherapy in esophageal cancer
Source: Phys Imaging Radiat Oncol. 2025 Jan 28;33:100717. doi: 10.1016/j.phro.2025.100717 (PMC11840183; doi:10.1016/j.phro.2025.100717)
Supplement: Supplementary Data 1 [file mmc1.pdf]

Supplementary Materials A – Tumor characteristics

Table A1. *Tumor characteristics of the nine patients included in the in-silico assessment and the first seven patients clinically treated with the ATS-lite workflow.*

Characteristic	In-silico assessment patients	Clinical ATS-lite patients
**No. of patients**	9	7
**Histology**		
**Adenocarcinoma**	5	5
**Squamous cell carcinoma**	4	2
**Tumor location**		
**Mid-esophagus**	2	2
**Distal-esophagus**	5	4
**Gastroesophageal junction**	2	1
**Tumor classification**		
**cT2**	1	2
**cT3**	7	4
**cT4**	1	1
**Nodal classification**		
**N0**	6	4
**N1**	3	2
**N2**	0	1

Supplementary Materials B – Quality assurance on interfraction dose accumulation

Methods

Quality assurance (QA) on the registration result and deformation vector field was performed using visual inspection, the structural similarity index [1,2] and Jacobian determinant [3]. Structural similarity is used to compare the image similarity between the pre-treatment and registered fraction scan. We evaluated the mean value on all voxels of the CTV and a surrounding band of 10 voxels. For the Jacobian determinant, we determined the percentage of voxels with a value below 0 and the percentage of voxels with a value above 2, as recommended in [3], on the CTV and its surrounding.

In addition, we assessed the robustness of the target coverage for small geometric uncertainties associated with the interfraction dose accumulation. To achieve this, the pre-treatment CTV (CTV_{ORG}) was expanded by 0.6 x 0.6 x 0.0 mm^3, 1.2 x 1.2 x 0.0 mm^3, and 1.8 x 1.8 x 2.0 mm^3, considering the voxel spacing of 0.6 x 0.6 x 2 mm^3. The coverage of these expanded CTV_{ORG} contours by the total treatment dose was then evaluated for both the ATS and ATS-lite workflows.

Results

For the in-silico assessment patients, the mean structural similarity improved with a factor of 1.6 from 0.37 after rigid alignment to 0.59 after non-rigid registration. The percentage of voxels with a Jacobian determinant below zero (indicating an estimation of anatomically implausible tissue folding) was 0.00%, and the percentage of voxels with a value above 2 (indicating significant expansion, affecting the warped dose) was 0.08%. Visual inspection confirmed the quality of the registrations, with no errors affecting the target coverage assessment.

For the clinical ATS-lite patients, the mean structural similarity improved with a factor of 1.8 from 0.37 after rigid alignment to 0.66 after non-rigid registration. The percentage of voxels with a Jacobian determinant below zero was 0.00%, and the percentage of voxels with a value above 2 was 0.03%.

After expanding the CTV_{ORG} with a margin of 1.8 x 1.8 x 2.0 mm^3, the total treatment dose of the ATS-lite workflow still achieved a coverage of V95% > 99.1% and V90% > 99.9% for all patients (Figure B1). These results demonstrate that the assessed target coverage is robust against small geometric uncertainties potentially introduced during interfraction dose accumulation.

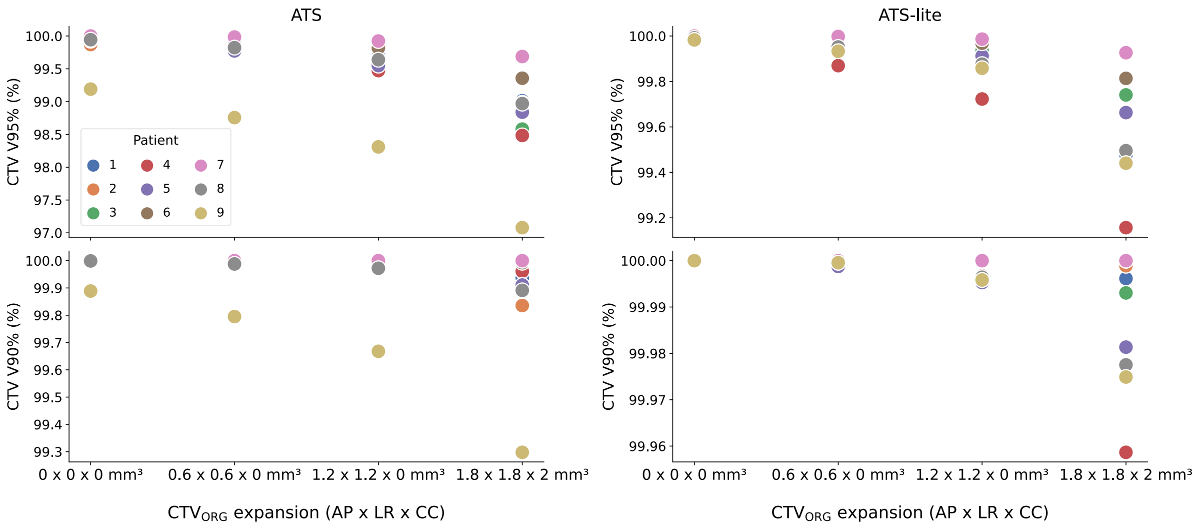

Figure B1. *Target coverage by the total treatment dose for the ATS and ATS-lite workflows after gradual expansion of the pre-treatment clinical target volume (CTV$_{ORG}$).*

References Supplementary Materials B

[1] Wang Z, Bovik AC, Sheikh HR, Simoncelli EP. Image quality assessment: from error visibility to structural similarity. IEEE Transactions on Image Processing 2004;13:600–12. https://doi.org/10.1109/TIP.2003.819861.

[2] Bosma LS, Zachiu C, Denis de Senneville B, Raaymakers BW, Ries M. Technical note: Intensity-based quality assurance criteria for deformable image registration in image-guided radiotherapy. Med Phys 2023;50:5715–22. https://doi.org/https://doi.org/10.1002/mp.16367.

[3] Bosma LS, Hussein M, Jameson MG, Asghar S, Brock KK, McClelland JR, et al. Tools and recommendations for commissioning and quality assurance of deformable image registration in radiotherapy. Phys Imaging Radiat Oncol 2024;32:100647. https://doi.org/https://doi.org/10.1016/j.phro.2024.100647.

Supplementary Materials C – ATS versus ATS-lite OAR dose volume histogram metrics

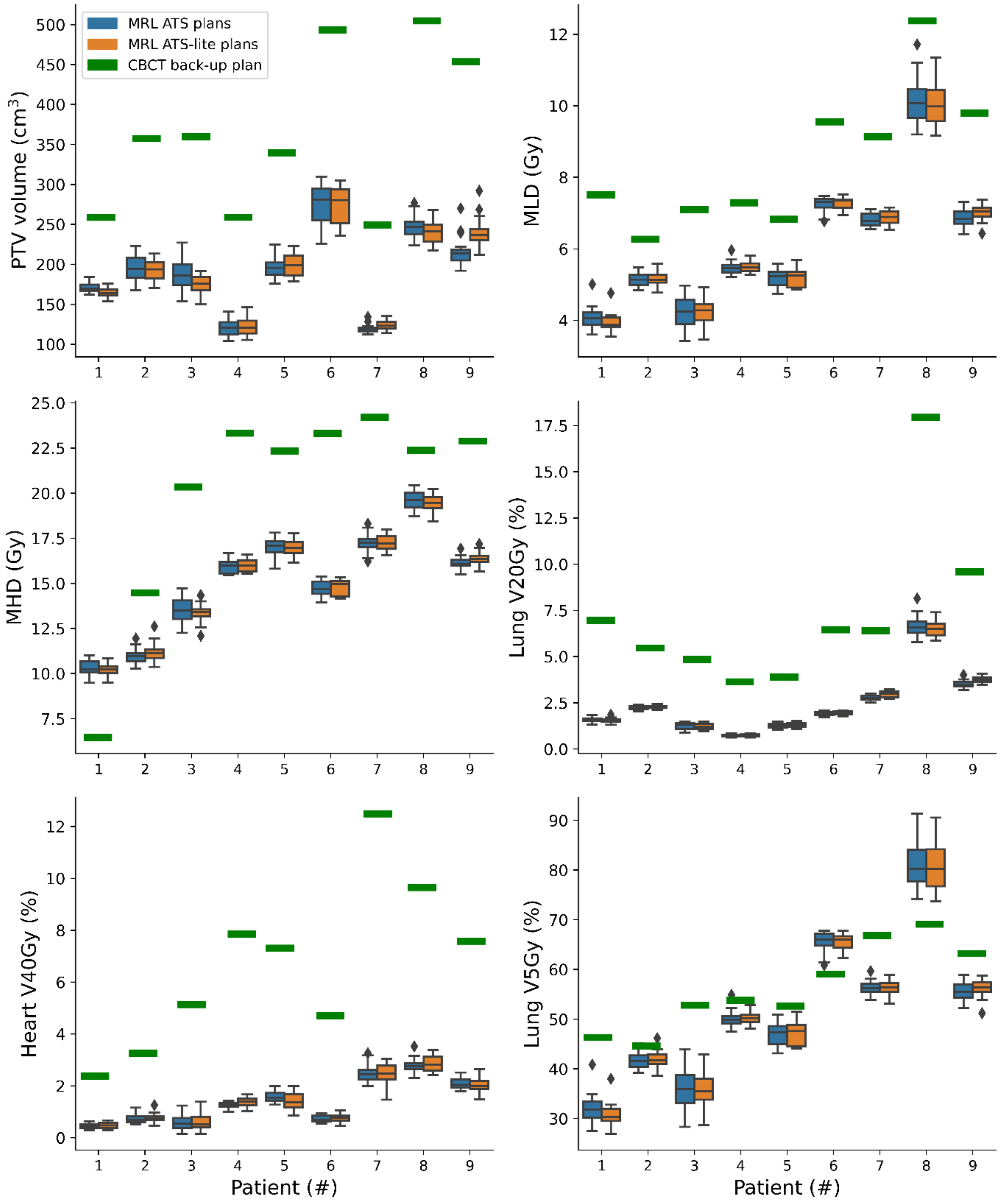

***Figure C1**: Planning target volume (PTV) sizes and organs-at-risk dose volume histogram metrics of each intrafraction accumulated ATS (blue) and ATS-lite (orange) dose distribution in the in-silico assessment. Additionally, the metrics for a clinically approved CBCT-guided back-up plan with a 10 mm isotropic PTV margin are also shown to illustrate the dosimetric benefits of the ATS-lite workflow compared to conventional CBCT-guided treatment plans. MHD: mean heart dose. MLD: mean lung dose.*

The conformality index (CI) was used to evaluate the spatial precision of the total treatment dose for both workflows. The CI for a dose of 39.33 Gy was calculated according to the equation below, and quantifies how well the prescribed dose conforms to the target volume. A CI of 1 indicates perfect conformality with no prescribed high dose outside the target volume. Since all total treatment dose distributions achieved complete target coverage, CI values below 1 indicate unnecessary exposure of surrounding healthy tissue. The quality of the total treatment dose distributions of the ATS and ATS-lite workflow are comparable (Figure C2).

$$Conformality\ index = \frac{(CTV_{ORG} \cap\ 39.33\ Gy)^2}{(CTV_{ORG}) * (39.33\ Gy)}$$

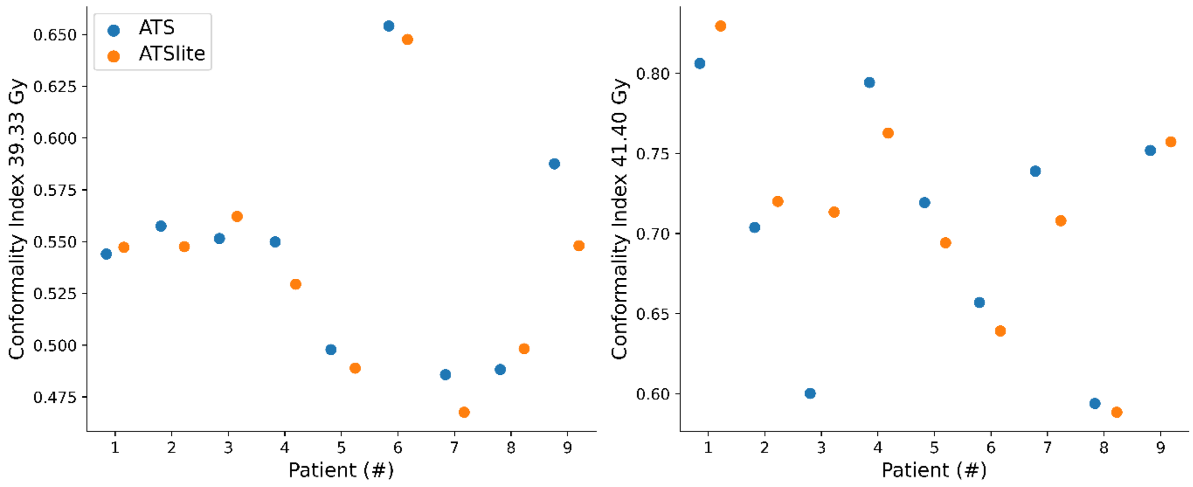

***Figure C2**: Conformality indices for the total treatment dose of the ATS (blue) and ATS-lite (orange) workflow with the pre-treatment clinical target volume (CTV_{ORG}) for thresholds of 39.33 Gy (left) and 41.40 Gy (right).*

Supplementary Materials D – Individual fractions with insufficient target coverage

Except for patient 3, the ATS-lite workflow resulted in insufficient coverage of the clinical-ATS CTV (CTV_{ATS}) in only four fractions. Upon closer inspection, it was found that underdosage in individual fractions was primarily due to large variation in the CTV_{ATS} contours, which served as ground truth in this analysis. Regions with underdosing predominantly occurred where the radiation oncologist had increased the CTV_{ATS} relative to the CTV_{ORG} on the reference MRI, from which contour propagation was performed. The substantial variation in the CTV_{ATS} contours raised doubts about the 'ground truth' labelling in these fractions, and suggests that inadequate tumor coverage was not attributable to the ATS-lite workflow. This suggestion is supported by the complete coverage of the CTV_{ORG} by the total accumulated dose of the ATS-lite workflow. In this Supplementary Materials, we provide detailed illustrations of the underdosage areas and the variation in the CTV_{ATS} contours for each fraction with insufficient target coverage.

Figure D1 depicts volumetric and length differences between CTV_{ORG} and CTV_{ATS} in relation to the CTV_{ATS} V95% and CTV_{ATS} V90%. The CTV_{ATS} volume or length was increased relative to the CTV_{ORG} for most fractions with insufficient coverage of the CTV_{ATS}. In addition to this quantitative analysis, we provide detailed illustrations of the underdosage areas and the variations in the CTV_{ATS} contours for each fraction with inadequate target coverage.

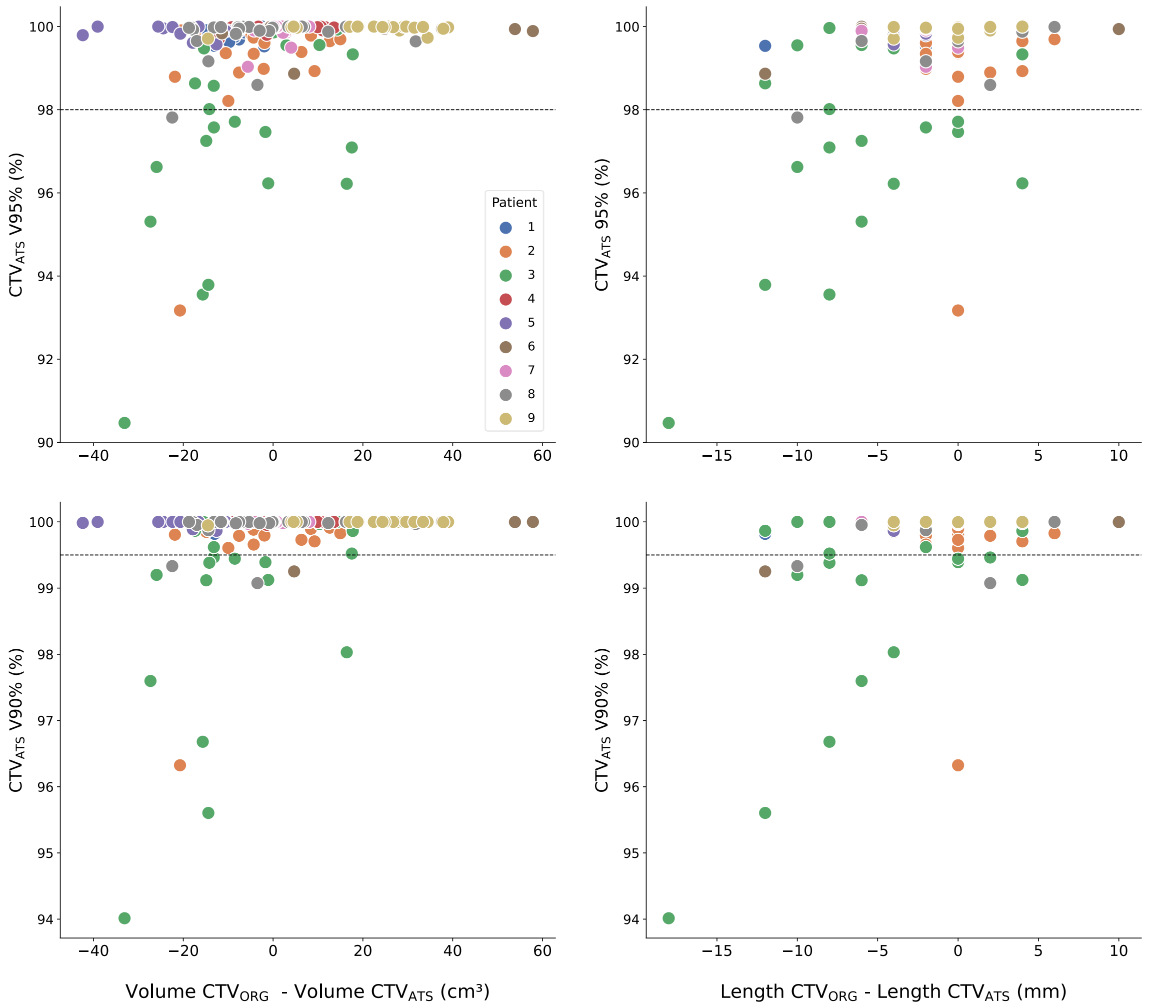

Figure D1. *Coverage of the manually corrected CTV from the clinical ATS workflow* (CTV_{ATS}) *in individual fractions, including* CTV_{ATS} *V95% (top) and* CTV_{ATS} *V90% (bottom), shown in relation to volume (left) and length (right) differences between the CTV on the reference MRI used for propagation* (CTV_{ORG}) *and the* CTV_{ATS}. *Black dotted lines indicate the thresholds for adequate target coverage, defined as* CTV_{ATS} *V95% > 98% and* CTV_{ATS} *V90% > 99.5%.*

Patient 2

In patient 2, one outlier fraction resulted in insufficient target coverage with a CTV_{ATS} V95% of 93.2% and V90% of 96.3%. Closer inspection revealed an increase of the CTV_{ATS} contour in the distal and proximal part of the CTV, corresponding to the underdosed areas (Figure D2).

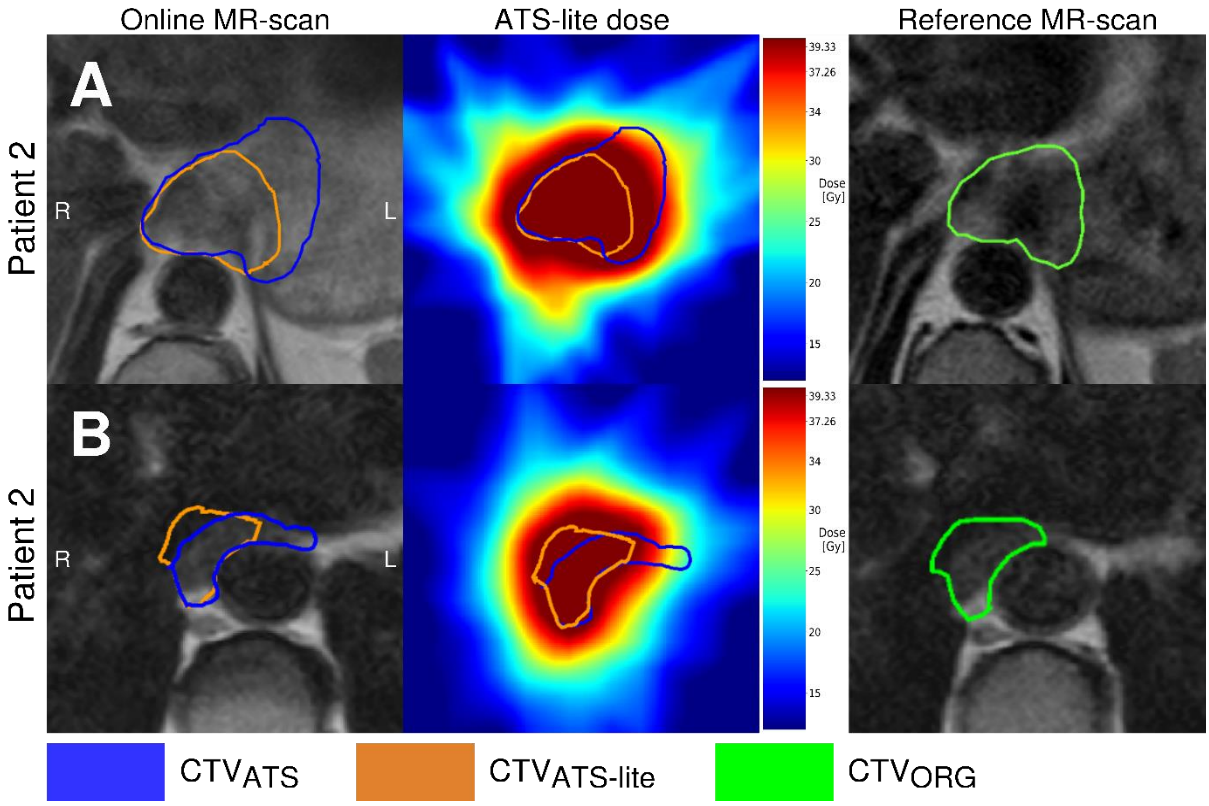

Figure D2. *Fraction with insufficient target coverage in patient 2, showing underdosage in the distal (A) and proximal (B) parts of the clinical-ATS CTV* (CTV_{ATS}, *blue). In both regions, a substantial increase of the* CTV_{ATS} *in the online MR-scan (left) compared to the pre-treatment CTV (*CTV_{ORG}*, green) on the reference MR-scan (right) can be observed. These enlargements resulted in underdosage of the* CTV_{ATS} *by the intrafraction accumulated ATS-lite dose distribution (middle).*

Patient 6

For patient 6, all fractions achieved high coverage of the CTV_{ATS} (V95% ≥ 99.9% and CTV V90% = 100%), except for one fraction with a CTV_{ATS} V95% of 98.7% and CTV_{ATS} V90% of 99.3%. In this fraction, the cranio-caudal CTV_{ATS} length was 166 mm, compared to 154 mm of the CTV_{ORG} on the reference MR-scan (Figure D3). The 12 mm increase is clinically unrealistic and was not observed in other fractions, where the maximum increase was 6 mm. Figure D3 also shows that the CTV_{ATS} contour on the online MR-scan extends into the stomach, whereas the CTV_{ORG} did not on the reference MR-scan.

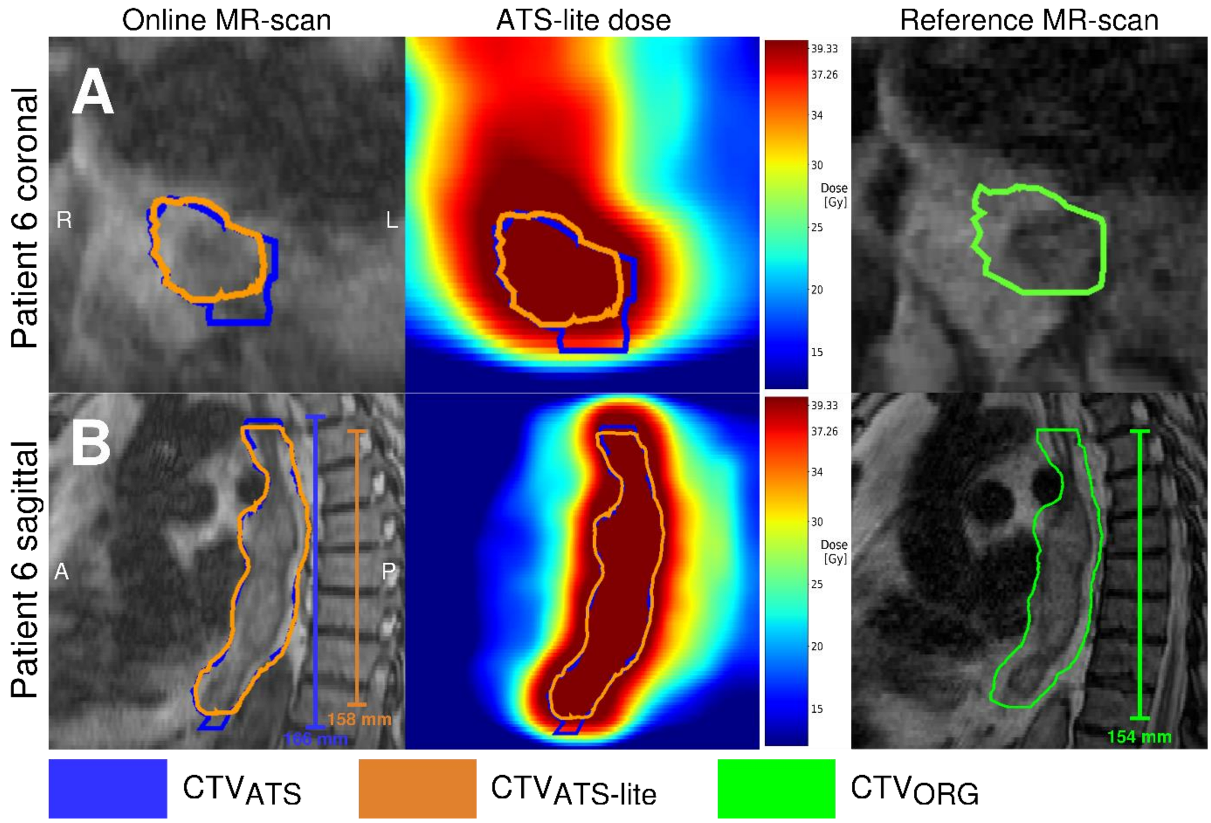

Figure D3. *Fraction with insufficient target coverage in patient 6. The longitudinal* CTV_{ATS} *length (blue) on the online MR-scan (left) is enlarged relative to the* CTV_{ORG} *(green) on the reference MR-scan (right). Consequently, the ATS-lite intrafraction accumulated dose resulted in underdosage of the* CTV_{ATS} *in the stomach extension (middle).*

Patient 8

Two fractions from patient 8 resulted in insufficient CTV_{ATS} coverage, with CTV_{ATS} V95% values of 98.6% and 97.8%, and CTV_{ATS} V90% values of 99.1% and 99.3%, respectively. In both fractions, the CTV_{ATS} included peri-esophageal fat tissue around the aorta, which was not included in the CTV_{ORG} on the reference MR-scan (Figure D4, row A and C). This inclusion is understandable, as patient 8 was undergoing definitive chemoradiation, and the thickness of the fatty tissue had increased, possibly due to chemotherapy. However, in other fractions, the peri-esophageal fat tissue around the aorta was thinner and therefore not included in the CTV contour. From a radiation biology perspective, it is unreasonable to include tissue in the CTV in only a few fractions. Therefore, it is important to define the tissue to be included in the pre-treatment phase and to adhere to this definition consistently throughout the treatment.

In the second fraction, the CTV_{ATS} extended into the stomach contents, which are not part of the target volume (Figure D4, row B). On the other hand, the ventral part of $CTV_{ATS\text{-}lite}$ contour should have extended slightly further into the stomach when comparing to the CTV_{ORG} on the reference MR-scan.

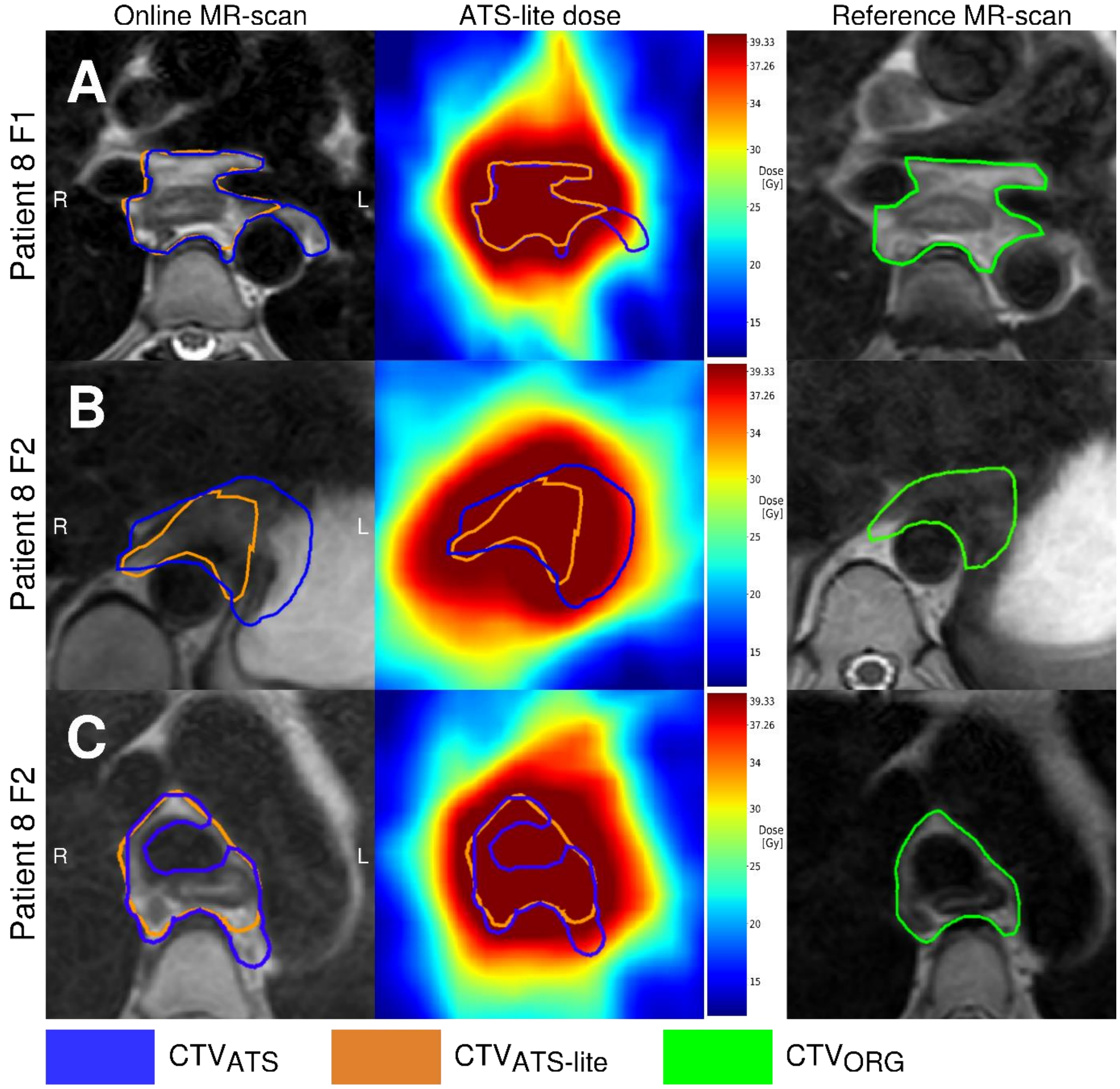

Figure D4. *Two fractions with insufficient coverage of the* CTV_{ATS} *in patient 8. In both fractions, additional peri-esophageal fat tissue around the aorta was included in the* CTV_{ATS} *(blue) compared to the* CTV_{ORG} *on the reference MR-scan (right) (row A and C). Additionally, stomach contents were included in the* CTV_{ATS} *in one fraction (row B).*

Patient 3

In patient 3, the variation in CTV_{ATS} contours was significantly larger and more frequent compared to the other patients (Figure D1). This discrepancy is most likely also attributed to the involvement of a larger group of radiation oncologists, including less experienced observers, in contouring patient 3. Variation in the CTV_{ATS} contours was primarily observed in the longitudinal length (Table D1). Additionally, patient 3 exhibited substantial intrafraction motion, primarily in cranio-caudal direction [4]. The combination of these two factors led to inadequate coverage of the CTV_{ATS} in 57% of the fractions.

Table D1. *Increased longitudinal length of the* CTV_{ATS} *of each fraction compared to the length of the* CTV_{ORG} *on the reference MR-scan. The slice thickness of the MR-scans was 2 mm.*

Fraction #	1	2	3	4	5	6	7	8	9	10	11	12
Increased CTV_{ATS} length (mm)	-2	4	6	8	12	6	12	18	8	0	2	-4
Fraction #	13	14	15	16	17	18	19	20	21	22	23	
Increased CTV_{ATS} length (mm)	0	10	8	10	4	4	-4	6	-2	8	2	

References Supplementary Materials D

[4] Boekhoff MR, Lagendijk JJW, van Lier ALHMW, Mook S, Meijer GJ. Intrafraction motion analysis in online adaptive radiotherapy for esophageal cancer. Phys Imaging Radiat Oncol 2023;26:100432. https://doi.org/10.1016/j.phro.2023.100432.
